# Immunophenotypes of macular corneal dystrophy in India and correlation with mutations in *CHST6*

**Published:** 2009-02-09

**Authors:** Afia Sultana, Gordon K. Klintworth, Eugene J-M.A. Thonar, Geeta K. Vemuganti, Chitra Kannabiran

**Affiliations:** 1 Kallam Anji Reddy Molecular Genetics Laboratory, Hyderabad Eye Research Foundation, L.V. Prasad Eye Institute, Hyderabad, India; 2 Department of Ophthalmology and Pathology, Duke University Medical Center, Durham, NC; 3 Department of Biochemistry and Internal Medicine, Rush Presbyterian-St. Luke’s Medical Center, Chicago, IL; 4 Ophthalmic Pathology Service, Hyderabad Eye Research Foundation, L.V. Prasad Eye Institute, Hyderabad, India

## Abstract

**Purpose:**

To determine the immunophenotypes of macular corneal dystrophy (MCD) in Indian patients and to correlate them with mutations in the carbohydrate 6-sulfotransferase (*CHST6*) gene.

**Methods:**

Sixty-four patients from 53 families with MCD that were previously screened for mutations in *CHST6* were included in an immunophenotype analysis. Antigenic keratan sulfate (AgKS) in serum as well as corneal tissue was evaluated in 31 families.  Only cornea was evaluated in 11 families, and only serum was evaluated in 11 families. AgKS was detected in formalin-fixed, paraffin-embedded corneal sections by immunohistochemistry and in serum by ELISA using a monoclonal antibody against sulfated forms of KS in patients with MCD as well as normal controls.

**Results:**

Analysis of corneal and/or serum AgKS disclosed MCD type I (27 families), MCD type IA (5 families), and MCD type II (3 families) in the cases studied. An additional 10 families were either MCD type I or MCD type IA since only serum AgKS data were available. Seven families manifested atypical immunophenotypes since the corneal AgKS expression was either of MCD type I or MCD type IA, but serum AgKS levels ranged from 19 ng/ml to 388 ng/ml. More than one immunophenotype was detected amongst siblings in two families. Each immunophenotype was associated with mutational heterogeneity in *CHST6*.

**Conclusions:**

MCD type I was the predominant immunophenotype in the Indian population studied followed by MCD type IA and then MCD type II. We detected further immunophenotypic heterogeneity by finding atypical patterns of AgKS reactivity in a subset of families. There were no simple correlations between immunophenotypes and specific mutations in *CHST6*, suggesting that factors other than *CHST6* mutations may be contributing to the immunophenotypes in MCD.

## Introduction

Macular corneal dystrophy (MCD; OMIM 217800) is an autosomal recessive disorder characterized clinically by bilateral diffuse clouding of the corneal stroma and the presence of grayish-white, punctate opacities especially in the superficial cornea. Histologically, the cornea in MCD is characterized by the accumulation of extracellular deposits in the stroma and Descemet membrane as well as by intracellular storage of similar material in the keratocytes and corneal endothelium. The deposits stain with Alcian blue and other histochemical methods for glycosaminoglycans (GAGs) [1]. Biochemical studies based on organ cultures of corneas as well as analyses of serum in patients with MCD suggested that the basic defect in MCD lies in a sulfotransferase specific for sulfation of keratan sulfate (KS) proteoglycan [2-7]. Molecular genetic studies on MCD led to the mapping of the MCD gene [8] and subsequently to the identification of the carbohydrate 6-sulfotransferase (*CHST6*) gene, which codes for corneal N-acetyl glucosamine 6-sulfotransferase, as the cause for MCD [9].

Although MCD is clinically homogeneous, three immunophenotypes have been recognized based on the reactivity of the cornea and serum in patients with MCD to a monoclonal antibody, 5D4, which is specific for highly sulfated epitopes on KS [10-13]. MCD type I is characterized by the absence or very low levels of antigenic keratan sulfate (AgKS) in the cornea and serum. MCD type II is characterized by the presence of normal or slightly reduced levels of AgKS in serum and an immunohistochemical reactivity for AgKS in the corneal stroma, keratocytes, Descemet membrane, and endothelial cells [10]. In addition, a third immunophenotype (MCD type IA) is characterized by an absence or very low level of serum AgKS accompanied by detectable AgKS reactivity in keratocytes [11]. The deposits in MCD type I were further shown to be unsulfated by *Erythrina crystagalli* agglutinin labeling, which recognizes unsulfated keratan sulfate proteoglycan (KSPG) [13]. Akama and coworkers [9] suggested that MCD types I and II were distinguishable by the nature of *CHST6* mutations. They observed that MCD type II was associated with a deletion or rearrangement of the upstream region of *CHST6* and proposed that MCD type II is characterized by an absence of coding region mutations in one or both alleles [9]. However, deviations from this pattern have been observed in subsequent studies [14,15]. Despite the fact that the immunophenotypes of MCD do not seem to be clinically relevant, we wanted to determine the prevalence of different MCD subtypes in Indian patients and to evaluate the relation of *CHST6* mutations with the aforementioned immunophenotypes in a series of 53 Indian families with MCD in whom mutational data on *CHST6 *was available [16,17].

## Methods

The study had the approval of the Institutional Review Board of the L. V. Prasad Eye Institute (Hyderabad, India) and conformed to the tenets of the Declaration of Helsinki. Informed consent was obtained from all participants, and 4 ml of peripheral blood was collected from each participant for serum analysis of AgKS. The diagnosis of MCD was based on the distinct clinical features (as described previously [16]), and in 48 patients this was confirmed by a histopathologic examination of the excised corneal buttons obtained at penetrating keratoplasty. All patients included in the present study were from the cohort previously screened for mutations in *CHST6* [16,17].

Immunohistochemistry was performed on 4 μ thick sections of paraffin-embedded formalin-fixed corneas from 48 MCD patients from 42 families (55 total corneas). Monoclonal primary antibody directed specifically against sulfated epitopes present on KS (1/20/5-D-4 mouse IgG; ICN Pharmaceuticals Inc., Irvine, CA) was used for detection of AgKS in the cornea. The secondary antibody used was biotinylated anti-mouse IgG (DakoCytomation, Glostrup, Denmark). Detection was done by the avidin-biotin complex immunoperoxidase technique in which avidin-conjugated peroxidase binds to the biotinylated antibody followed by detection of peroxidase after the addition of a suitable substrate, 3,3′-diaminobenzidine tetrahydrochloride (DAB; Sigma-Aldrich, St. Louis, MO) [18]. The primary antibody was used at a dilution of 1:700, which was determined as the optimum dilution by testing different antibody dilutions using corneas from positive (normal) controls that included patients with congenital hereditary endothelial dystrophy 2 (CHED2) or granular corneal dystrophy (GCD) in which KS is expected to be normal. As a negative control, the primary antibody was omitted and phosphate-buffered saline was used. Interpretations of immunohistochemical assays were made independently by four investigators, each masked to the findings of the others.

### Estimation of serum AgKS

Serum AgKS levels were determined in 51 MCD patients from 42 families and five unaffected relatives from 5 families by the enzyme-linked immunosorbent assay (ELISA) using the 1/20/5-D4 anti-KS monoclonal antibody as described previously [19]. Serum AgKS levels that were less than or equal to 8 ng/ml were interpreted as MCD types I/IA and levels of 100 ng/ml or above as normal or MCD type II.

## Results

### Immunophenotypes

**Figure 1:**
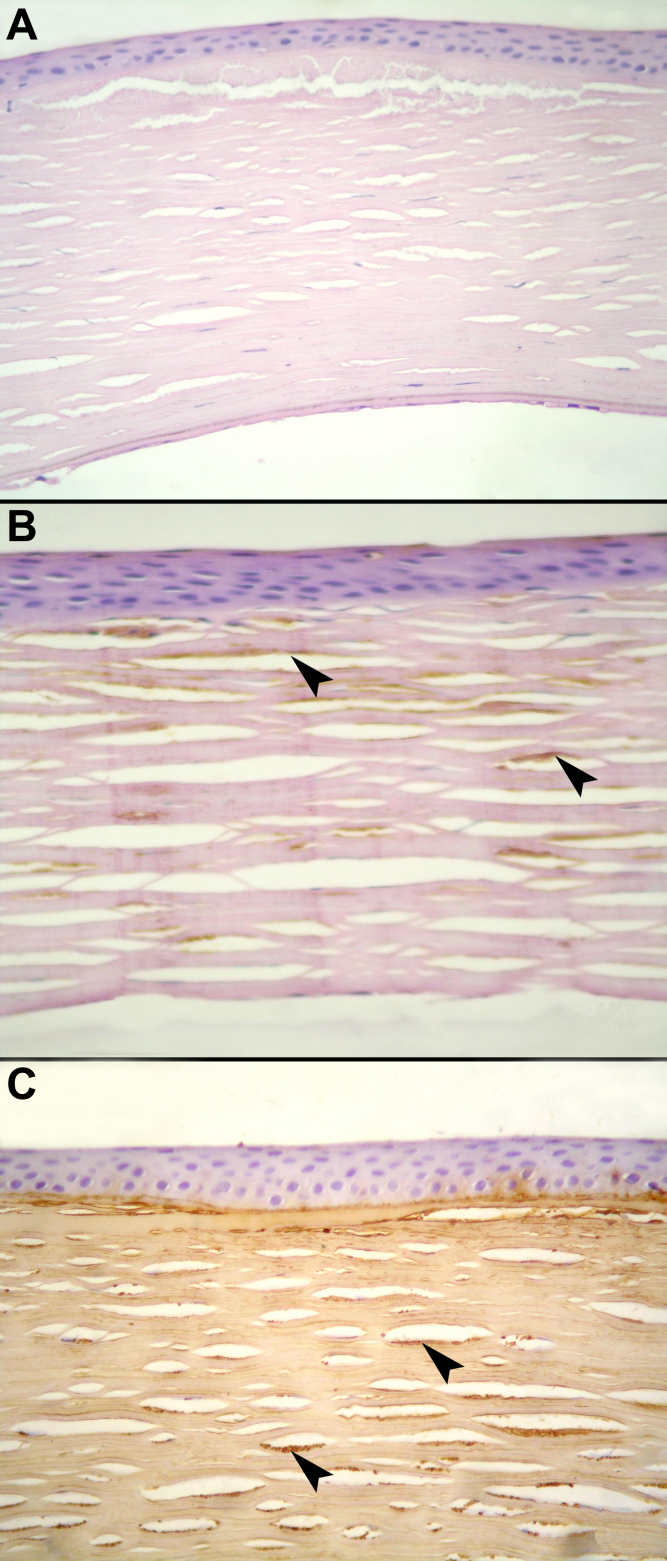
Immunophenotypes of MCD. Light microscopic images of representative corneas show different immunophenotypes of MCD (stained with diaminobenzidine [DAB], counterstained with hematoxylin-eosin). **A**: A cornea with the absence of detectable AgKS is shown (MCD type I; DAB; 40X). **B**: A cornea is shown with reactivity to AgKS only in stromal keratocytes (MCD type IA; DAB; 100X). **C**: A cornea is shown with AgKS detected throughout the stroma (MCD type II; DAB; 100X). Arrowheads point to the deposits in stromal keratocytes.

Fifty-three families with MCD (64 patients) were evaluated for the AgKS reactivity of their corneal tissue and/or serum. Representative corneas showing the three different immunophenotypes are illustrated in Figure 1A-C. Levels of AgKS were assessed in both the cornea and serum of 31 families (35 patients) and in either cornea or serum in 22 families.

Of these, AgKS levels corresponding to MCD type I were found in 31 patients from 27 families (patients 1–31; Table 1) based on analyses of both the cornea and serum in at least one affected individual (20 families) and of only the cornea (7 families). More than one individual was available for this study in two families, and different members within each family showed similar patterns of AgKS (patients 21–22 and 28–31; Table 1).

**Table 1:** Immunophenotypes and associated mutations in patients with MCD.

**Patient number**	**Reactivity to KS antibody in**		**Serum AgKS (ng/ml)**	**Type of mutation**	**Mutation**	**MCD type**
	**Stroma**	**Keratocytes**				
1	-ve	-ve	<4	Frameshift	p.Ser32GlnfsX36	I
2	-ve	-ve	<2	Missense	p.Gly52Asp	I
3	-ve	-ve	Not done	Missense	Ser53Leu	I
4	-ve	-ve	<4	Missense	p.Phe107Ser	I
5	-ve	-ve	<4	Missense	p.Phe107Ser	I
6	-ve	-ve	Not done	Nonsense	p.Trp123X	I
7	-ve	-ve	<4	Nonsense	p.Cys153X	I
8^#^	-ve	-ve	<2	Insertion	p.Arg195–196ins	I
9	-ve	-ve	<4	Missense	p.Pro204Gln	I
10^#^	-ve	-ve	<2	Frameshift	p.Arg205TrpfsX176	I
11	-ve	-ve	6	Insertion	p.Trp219–220ins	I
12	-ve	-ve	<4	Insertion	p.Trp219–220ins	I
13	-ve	-ve	<4	Insertion	p.Trp219–220ins	I
14	-ve	-ve	<4	Nonsense	p.Glu347X	I
15	-ve	-ve	5	Frameshift	p.Val6ProfsX55	I
16	-ve	-ve	6	Missense	p.Ser54Phe	I
17	-ve	-ve	7	Frameshift	p.Phe60LeufsX10	I
18	-ve	-ve	8	Frameshift	p.Phe60LeufsX10	I
19	-ve	-ve	Not done	Missense	Cys165Ser	I
20	-ve	-ve	Not done	Missense	Cys165Trp	I
21^A^	-ve	-ve	<2	Missense	p.Pro204Arg	I
22^A^	Not done	Not done	<2	Missense	p.Pro204Arg	I
23	-ve	-ve	<2	Insertion	p.Trp219–220ins	I
24	-ve	-ve	Not done	Insertion	p.Trp219–220ins	I
25	-ve	-ve	<2	Missense	p.Asp221Tyr	I
26	-ve	-ve	Not done	Missense	p.Arg334Cys	I
27	-ve	-ve	Not done	Frameshift	p.His335CysfsX27	I
28^B^	-ve	-ve	<4	Frameshift	p.His335CysfsX27	I
29^B^	-ve	-ve	<2	Frameshift	p.His335CysfsX27	I
30^B^	Not done	Not done	<2	Frameshift	p.His335CysfsX27	I
31^B^	Not done	Not done	<2	Frameshift	p.His335CysfsX27	I
32^C^	-ve	+ve	<4	Missense	p.[Ser98Trp]+[Phe107Ser]	IA
33^C^	-ve	+ve	<4	Missense	p.[Ser98Trp]+[Phe107Ser]	IA
34	-ve	+ve	<4	Missense	p.Phe121Ser	IA
35	-ve	+ve	<4	Insertion	p.Trp219–220ins	IA
36	-ve	+ve	Not done	Missense	p.Asp221Glu	IA
37^#D^	-ve	+ve	Not done	Missense	p.Asp221Glu	IA
38^#D^	-ve	+ve	Not done	Missense	p.Asp221Glu	IA
39	+ve	+ve	not done	Frameshift	p.[Phe67SerfsX3]+[=]	II
40	+ve	+ve	not done	Nonsense +missense	p.[Trp2X;Leu3Met]+ [Trp2X;Leu3Met]	II
41^E^	Not done	Not done	105	-	No CHST6 mutation	II?
42^E^	Not done	Not done	42	-	No CHST6 mutation	II/atypical?
43	Not done	Not done	<4	Missense	p.Gly52Asp	I/IA
44	Not done	Not done	<4	Frameshift	p.Phe60LeufsX10	I/IA
45	Not done	Not done	<4	Nonsense	p.Gly309X	I/IA
46	Not done	Not done	<4	Missense	p.[Val56Arg]+ [Ser167Phe]	I/IA
47	Not done	Not done	<4	Missense	p.Ala73Thr	I/IA
48	Not done	Not done	8	Nonsense	p.Trp123X	I/IA
49	Not done	Not done	<4	Missense	p.Ser131Pro	I/IA
50^F^	Not done	Not done	<4	Frameshift+ Missense	[Gln182ArgfsX199]+ [Leu276Pro]	I/IA
51^F^	Not done	Not done	<4	Missense	p.Leu276Pro	I/IA
52	Not done	Not done	<2	Missense	p.Leu193Pro	I/IA
53	Not done	Not done	<2	Missense	p.Arg272Ser	I/IA
54	-ve	-ve	63	Nonsense	p.Gln18X	Atypical
55	-ve	+ve	95	Missense	p.Asp221Glu	Atypical
56	-ve	+ve	388	Missense	p.[Ser98Leu]+[=]	Atypical
57	-ve	-ve	33	Missense	p.Phe178Cys	Atypical
58	-ve	-ve	19	Missense	p.Arg202Ser	Atypical
59	-ve	+ve	142	Missense	p.Asp221Glu	Atypical
60^G^	-ve	-ve	61	Missense	p.[Ser210Phe]+[Asp221Glu]	Atypical
61^G^	-ve	+ve	Not done	Missense	p.[Ser210Phe]+[Asp221Glu]	IA?
62^H^	+ve	+ve	101	Missense	Asp221Tyr	II
63^H^	-ve	+ve	<4	Missense	Asp221Tyr	IA
64^H^	-ve	-ve	6	Missense	Asp221Tyr	I

Details of serum and corneal AgKS reactivity, associated *CHST6* mutations and corresponding MCD immunophenotypes are listed for all patients studied. Reactivity to AgKS in cornea is denoted as present (+ve) or absent (-ve). Mutations in the protein are as described earlier [16,17]. Members of the same family are denoted by letters A-H in superscripts. The sharp (hash mark ‘#’) indicates patients that had both corneas evaluated.

Five families (7 patients [# 32–38]; Table 1) had a pattern corresponding with MCD type IA as assessed by AgKS reactivity in the cornea or in both cornea and serum (Table 1). Two families each had two affected members (patients 32, 33 and patients 37,38) who showed the same immunophenotypes.

MCD type II was found in two unrelated patients (patients 39, 40) as indicated by corneal AgKS reactivity.

Eleven families (13 patients) were grouped into immunophenotypes based only on serum levels on AgKS since corneal tissues were unavailable. The serum AgKS levels in 10 families (11 patients) were less than 8 ng/ml, corresponding to MCD types I or IA (patients 43–53; Table 1) whereas in one family with two affected individuals (patients 41 and 42; Table 1), serum AgKS corresponded to MCD type II (105 ng/ml; patient 41) or possibly to an atypical pattern (patient 42; 42 ng/ml).

Apart from the three immunophenotypes mentioned above, there were seven families (7 patients) who showed AgKS reactivity that did not conform to any of the three types described and were therefore considered as atypical (patients 54–60; Table 1). These included four patients from four families with no detectable AgKS in the cornea (MCD type I pattern) and serum AgKS levels ranging from 19ng/ml to 63 ng/ml. The remaining three patients had detectable AgKS only in keratocytes (similar to MCD type IA) with serum AgKS levels ranging from 95 ng/ml to 388 ng/ml. Serum levels of AgKS in the three patients mentioned above are in the range of values for MCD type II.

**Figure 2:**
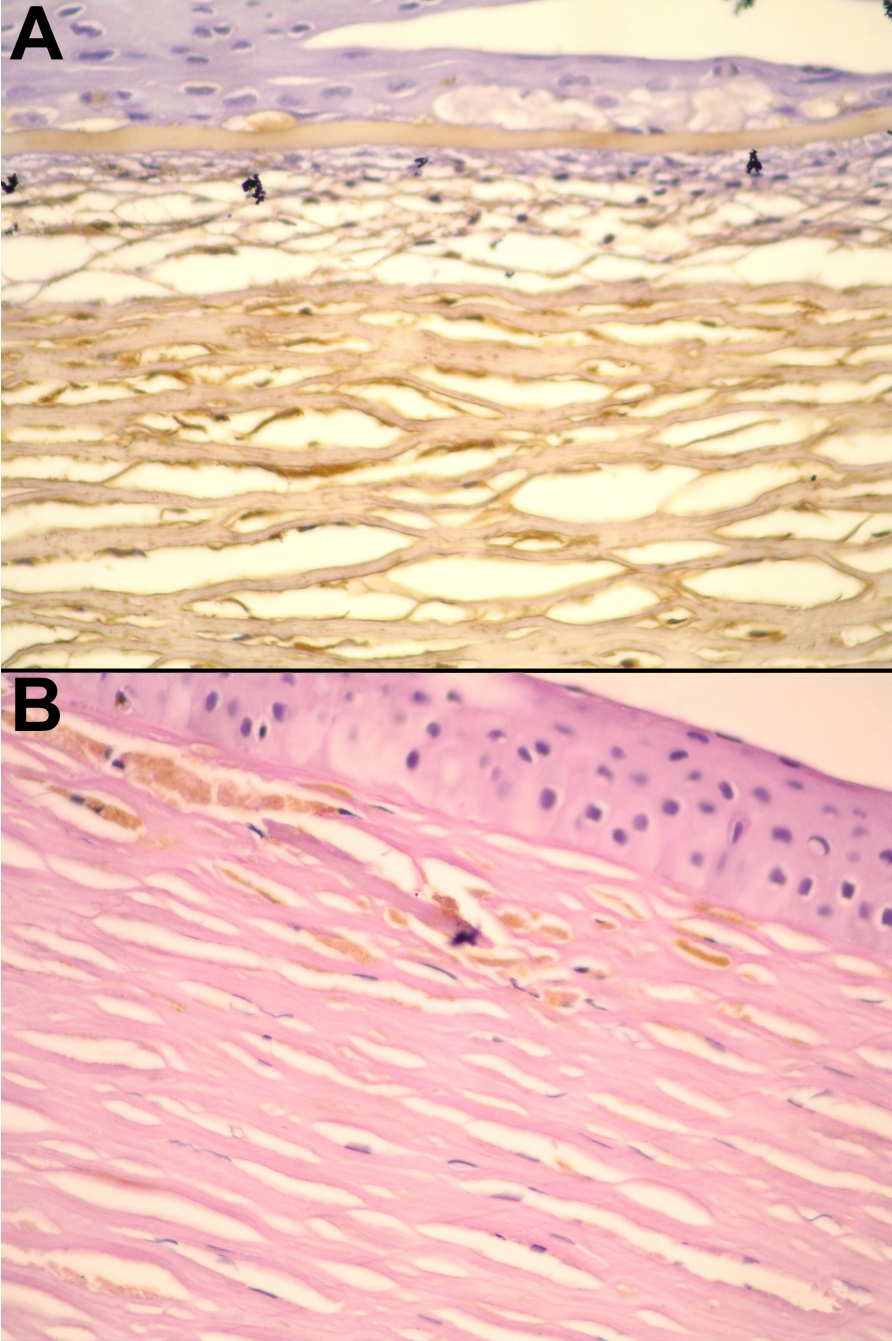
Different corneal immunophenotypes among affected members of a family with MCD. **A**: Corneal section from patient 62 having MCD type II, showing staining for AgKS throughout the stroma and in keratocytes. **B**: Corneal section from patient 63 from the same family, having MCD type IA. Staining for AgKS is seen only in corneal keratocytes, and is completely absent in the stroma (details in Table 1; stained with DAB, counterstained with hematoxylin-eosin and periodic acid Schiff, 400X).

Different immunophenotypes associated with the same *CHST6* mutation within a family were observed in two families (Table 1). Two members (patients 60, 61) from one family showed either an ‘atypical’ immunophenotype (patient 60, mentioned above) or a corneal immunoreactivity of MCD type IA (patient 61). In the second family, three members (patients 62–64; Table 1) were evaluated for both serum and corneal AgKS. The proband (patient 62) had the MCD type II phenotype whereas the two other members, the spouse and child (patients 63, 64),  had MCD types IA and I, respectively (Table 1). Photomicrographs of corneal sections of two members of this family are shown in Figure 2A,B.

### Correlations between mutations and immunophenotypes

#### MCD type I

Of the 27 families with MCD type I (Table 1), 10 different missense mutations were found in 11 families, three nonsense mutations in three families, five frameshift mutations in seven families, and two in-frame insertion mutations in six families. Homozygous mutations were identified in all families.

Similar mutational heterogeneity was observed in families grouped as either MCD type I or MCD IA based on serum Ag KS levels (Table 1).

#### MCD type IA

Of the five families classified as MCD type IA based on corneal AgKS, four families had three missense mutations with one mutation, Asp221Glu, in two families, and the fifth family had an in-frame insertion (Table 1).

#### MCD type II

MCD type II was found in association with a single heterozygous frameshift mutation (Phe67SerfsX3) in one family (patient 39), two homozygous mutations, Trp2Ter and Leu3Met, in one family (patient 40), and with no detectable mutations in another family (patient 41; Table 1).

#### Atypical immunophenotypes

Patients with ‘atypical’ immunophenotypes (Table 1) showed missense mutations (6 families), nonsense mutations (1 family), and no detectable mutation (1 family). Of these, one family (patient 56) had a single heterozygous mutation (Ser98Leu; Table 1).

## Discussion

This study examined immunophenotypes in MCD in relation to the mutational status of *CHST6* in MCD patients of Indian origin. The existence of distinct immunophenotypes of MCD has been well documented [10-12,20], and yet their biological or clinical significance remains unclear. Though a mutation in *CHST6*, presumed to lead to the absence of sulfation of KS precursors, is established as the cause of MCD, the presence of sulfated epitopes that are recognizable by the 5D4 monoclonal antibody in the cornea and/or serum of a subset of MCD patients suggests a further level of complexity in the pathogenesis of MCD. Understanding the molecular basis of the MCD immunophenotypes may lead to a better comprehension of the pathobiology of this inherited corneal disease.

The present study revealed that MCD type I is the most frequent immunophenotype in Indian patients. Taking into account those patients with their corneal and serum levels of AgKS evaluated, MCD type I, which was seen in 63% of patients (22/35 patients or 20/31 families), was the most common followed by MCD type IA, which was seen in 14% of patients (5/35 patients or 4/31 families), and then MCD type II, which was seen in 3% of patients (1/35 patients or 1/31 families). The predominance of MCD type I over other types is similar to that found in studies on MCD patients from various regions including Iceland, Saudi Arabia, southern India, and the United States [11,12,15,21,22]. A similar frequency of MCD types I and IA was reported in a study on German patients [23].

An unusual feature observed in the present study was that seven families showed ‘atypical’ immunophenotypes in which the values of serum AgKS ranged from 19 ng/ml to 388 ng/ml, although immunohistochemical analysis of these corneal sections showed little or no reactivity to AgKS, which would depict immunophenotypes MCD type I or MCD type IA. While the reasons for this are not currently understood, it may indicate further immunophenotypic heterogeneity in MCD. Complex immunophenotypes were also observed in a previous study by Iida-Hasegawa and coworkers [24] who found that a patient with serum AgKS corresponding to MCD type I had positive reactivity to AgKS in the stroma and extracellular accumulations. Moreover, patients with serum AgKS levels compatible with MCD type II in their study showed no stromal reactivity to AgKS on immunohistochemistry [24].

We found no clear-cut correlations between immunophenotypes and mutations in *CHST6*. Mutational heterogeneity was seen within each subtype of MCD. The same mutation was found to be associated with more than one immunophenotype. Of these, one mutation, Asp221Tyr, was found in patients from a single family with all three immunophenotypes while two other mutations, Trp219–220ins and Phe107Ser, were associated with MCD types I and IA. There were two recurrent mutations in our series, each found in six families, an insertion mutation, Trp219–220ins, and a missense mutation, Asp221Glu. Each was associated predominantly with one immunophenotype among the patients studied. Trp219–220ins was present in five families with MCD type I and in one family with MCD type IA (Table 1). The Asp221Glu mutation was predominantly found in association with a corneal phenotype of MCD type IA (patients 36–38, 55, 59, 61; Table 1) as well as with MCD type I in one patient (patient 60). However, serum AgKS values in three of these families showed atypical patterns (patients 55, 59, 60).

It is noteworthy that all three MCD subtypes were observed in one family, which was associated with the mutation, Asp221Tyr. The concordance between serum AgKS and corneal AgKS reactivity in each member of this family makes it unlikely that the variable immunoreactivity within the family is due to artifacts of detection. The presence of both MCD types I and II has also been noted previously in an Icelandic family, although with different but overlapping combinations of *CHST6* alleles [25,26].

There were very few patients with MCD type II in our series. A recurrent feature observed in different studies to date is that patients with MCD type II have no detectable mutations in the coding region and/or upstream region on one or both alleles of *CHST6* [14,15,21,22], although contradictory observations have been reported in some cases [14,26]. The predicted effect of mutations in the upstream (regulatory) regions and the absence of coding region mutations is that *CHST6* alleles in MCD type II would retain more enzymatic activity as compared to those responsible for MCD type I. We noted the occurrence of a single heterozygous mutation (patient 39; Table 1) or no detectable mutations in *CHST6* (patient 41; Table1) in two out of three families with MCD type II. It is worth noting that the other occurrence of a single heterozygous change in our series was in a patient having a corneal phenotype of MCD type IA but with normal levels of serum AgKS (patient 56; Table 1). Previous analysis in these patients with an absence of mutations in the coding region failed to detect mutations in the upstream region of *CHST6* [16,17]. Apart from these, one family with MCD type II in our study (patient 40; Table 1) had a double homozygous change of Trp2X and Leu3Met. An interesting possibility that needs to be explored further is that though a nonsense mutation Trp2X occurs at the second codon, a Leu3Met mutation in the next codon may generate a protein that is at least partially active due to re-initiation at codon 3 of the wild type protein.

In the background of the available literature and the results of the present study, the molecular basis of different immunophenotypes of MCD may not be readily explainable only on the basis of mutations in *CHST6*. KS is one of the major GAGs of the corneal stroma and plays an important role in corneal transparency. The variable presence of sulfated KS in cornea or serum of patients with *CHST6* mutations may be explained by a possible redundancy in KS sulfation activity both within the cornea and other tissues. Corneal N-acetyl glucosamine 6-sulfotransferase has been shown to efficiently catalyze the sulfation of endothelial mucin, which is the substrate for intestinal N-acetyl glucosamine sulfotransferase, thus showing that there is an overlap in activity with the intestinal sulfotransferase [27]. These possibilities, however, do not explain the absence of any clinical differences between MCD patients with different immunophenotypes. A biochemical evaluation of the enzymatic activities of proteins encoded by *CHST6* mutations associated with MCD type II versus mutations associated with MCD type I may reveal whether there are differences in activity between the two groups of mutants.

Data from the present study, which involved analysis of a large series of MCD patients from the same geographic origin, show complexity of immunophenotypes in MCD and suggest further immunophenotypic heterogeneity than previously described. The different immunophenotypes might be determined by factors other than or in addition to *CHST6* mutations.
